# Microscale Assemblies of Magnetic Nanoparticles Produced by Dip-Coating and Lift-Off With Dissolvable Templates

**DOI:** 10.1002/nano.70101

**Published:** 2025-12

**Authors:** Samuel D. Oberdick, Alexey V. Nazarov, Gary Zabow

**Affiliations:** 1 Department of Physics, University of Colorado, Boulder, Colorado, USA; 2 National Institute of Standards and Technology, Boulder, Colorado, USA

**Keywords:** magnetic nanoparticles, microfabrication, micropatterning

## Abstract

Rapid, low-cost methods for producing micropatterned arrays of nanoparticles can be used for many technological applications. This article describes a dip-coating technique for the fabrication of micrometer-scale assemblies of magnetic nanoparticles (MNPs). MNPs were deposited on top of dissolvable photoresist templates, which were created using photolithography in a cleanroom environment. When the templates were dissolved away, geometrically precise MNP arrays were left behind. The technique was used to produce micropatterned MNP composite arrays with engineered shapes, spacings, and positions.

## 1 | Introduction

Magnetic nanoparticles (MNPs) are nano-sized ferromagnetic crystals that have broad applications in fundamental physics research, biomedicine, materials science, and engineering [1–3]. For many applications, it is advantageous to guide the assembly of MNPs into ordered superstructures.

Many self-assembly and directed-assembly techniques for MNPs focus on relatively thin assemblies of particles. The formation of large area monolayer arrays, for example, has been studied for nanopatterning applications [4, 5]. Applied magnetic fields can be used to create chains of MNPs with high aspect ratios [6–8]. Chemically patterned surfaces can also be used to bind or precipitate MNPs within selective substrate regions, but these approaches produce thin patterns because the directed-assembly is localized to the surface [9, 10].

For some applications, directed-assembly techniques that produce thicker (micrometer-scale) MNP assemblies can be useful. MNP composites with multimicrometer thickness, for example, are being actively explored as core materials for high-frequency inductors [11–13]. For these applications, MNPs need to be directed into geometrically precise structures on top of patterned substrates. Electrophoretic deposition (EPD) can be used to create micrometer-thick films of MNPs, but this approach requires additional design considerations for integration of EPD electrodes [13–16]. Magnetophoretic deposition can also be used for the directed-assembly of micrometer-thick MNP assemblies [17]. However, this method requires specialized substrates with additional magnetic patterns [18].

Beyond high-frequency electronics and inductors, geometrically precise MNP assemblies with micrometer-scale thickness may be useful for applications in biomedicine and materials science, as well. For example, shaped MNP superstructures can be used as magnetic resonance imaging (MRI) sensors [19] and, also, magnetic materials with programmable magnetic anisotropy [20].

This article describes a method for creating MNP arrays with micrometer-scale thicknesses and well-defined shapes. The technique used dip-coating to deposit MNPs from solution on top of dissolvable templates. The templates, which were made of photoresist, were produced using photolithography in a cleanroom environment. MNP assemblies were fabricated using a lift-off approach—after MNPs were dip-coated onto the patterns, the photoresist was dissolved in acetone, leaving behind arrays of MNP superstructures on the substrate. The technique was used to create patterns with a lateral resolution of few micrometers. The method was compatible with multilayered fabrication schemes. In one demonstration, MNPs were deposited relative to underlying metal thin films or other structures. Also, anisotropic structures created using the technique showed an orientation-dependent magnetization.

## 2 | Experimental Section

### 2.1 | Materials

Ferrofluids (EMG 700) were acquired from Ferrotec. Acetone and isopropyl alcohol were acquired from Rocky Mountain Reagents. Photoresist and developers were supplied by the microfabrication facility at the National Institute of Standards and Technology (NIST) in Boulder, Colorado.

### 2.2 | Microfabrication

Photoresist templates were created using Megaposit SPR 220–3 photoresist and a Heidelberg MLA-150 maskless aligner. The photoresist was developed with Megaposit MF-26A developer. The gold thin film micropatterns were fabricated using electron beam evaporation and lift-off. Additional details are described in the Supporting Information (Section S1).

### 2.3 | Vibrating Sample Magnetometry (VSM)

Magnetometry was performed at room temperature using a Lake Shore 8607 VSM system.

### 2.4 | Optical Microscopy

Optical microscopy images were collected using a Nikon Eclipse LV100 microscope (main manuscript) and a Keyence VHX-7000 microscope (Figure S2, S3, and Video S1).

### 2.5 | Scanning Electron Microscopy (SEM)

SEM was performed using a Zeiss Sigma 300.

### 2.6 | Transmission Electron Microscopy (TEM)

TEM was performed using a FEI Tecnai T12 Spirit BT microscope (120 kV, LaB_6_). Images were analyzed using ImageJ to determine particle size distribution (Figure S1 and Section S2).

### 2.7 | Dip-Coater

Dip-coating was performed using a programmable Ossila Dip-Coater.

### 2.8 | Dip-Coating Experiments

The dip-coating apparatus had several programmable features that were used to control each deposition experiment. The immersion speed was the speed at which the substrate was lowered into the solution. The dwell time was the time that the substrate was submerged in the solution before being withdrawn. The withdrawal speed was the speed at which the substrate was removed from the solution. For key figures in the article, each of these parameters (insertion speed, dwell time, and withdrawal speed) is listed in Table 1.

During a typical deposition experiment, the substrate was mounted in the dip-coating apparatus. Then, a program was executed, which immersed that substrate at a programmed speed, submerged the substrate in solution (with no motion) for a programmed dwell time, and then withdrew the substrate from solution at a programmed speed. After the substrate was withdrawn from solution, the MNP-coated substrate was transferred to a hot plate, which was set to 70°C, for 3 min. This baking step was used to evaporate the remaining water from the deposited MNPs.

For all experiments, undiluted EMG 700 in its original concentration (5.8% by volume iron oxide) was used as the MNP solution. Note that this is a relatively high concentration for commercial MNP solutions. The equivalent concentration in mg magnetite (Fe_3_ O_4_) per ml of solution was ~ 3 ×10^2^ mg/ml (using a magnetite density of 5.24 g/ml [21]).

Withdrawal speeds of 0.05–2 mm/s produced micrometer-thick MNP depositions that could be reproducibly developed using the lift-off method. Slower speeds resulted in very thick films that overcoated the photoresist and prevented complete development of the patterns. The withdrawal speeds used in these experiments are consistent with the capillary regime of dip-coating, which can produce well-ordered micrometer-thick films of metal oxide nanoparticles from aqueous colloidal solutions [22].

The withdrawal speeds for this system were determined empirically. The fluid mechanical forces that govern dip-coating and evaporative assembly are complex and depend on template geometry, wetting/dewetting, and evaporative dynamics. Researchers should try several withdrawal speeds for each unique experimental system (combination of pattern geometry and colloidal suspension) to determine the best parameters for dip-coating.

### 2.9 | Development of MNP Micropatterns

The MNP-coated substrates were transferred to a beaker filled with acetone. The beaker was gently sonicated for 10–20 s, until most of the photoresist had been noticeably removed. Gentle sonication was achieved by connecting a bath ultrasonicator to a variable transformer. The transformer was set to 32% to 34%. The variable transformer was critical for producing uniform structures since ultrasonication with full power could delaminate or damage the MNP assemblies during development. After the first sonication step, the substrate was quickly transferred to a second glass of acetone and sonicated again (at 32% to 34% power) until the MNP micropatterns were fully developed. The substrate needed to be moved rapidly to the second beaker of acetone to prevent drying, which could potentially render regions undevelopable.

### 2.10 | Additional Notes

For researchers interested in trying the technique, two variables were found to be critically important for fabricating reproducible structures. First, for the ferrofluid used in this article (Ferrotec, EMG 700), the micropatterns developed best in acetone if the ferrofluid was used shortly after it had been delivered (within a 3-month timeframe). Secondly, micrometer-thick films were produced consistently if the ferrofluid was used with its original concentration. Dilutions of the stock solution produced thinner films.

## 3 | Results

### 3.1 | Fabrication Process and Patterned Assemblies of MNPs

**FIGURE 1 |:**
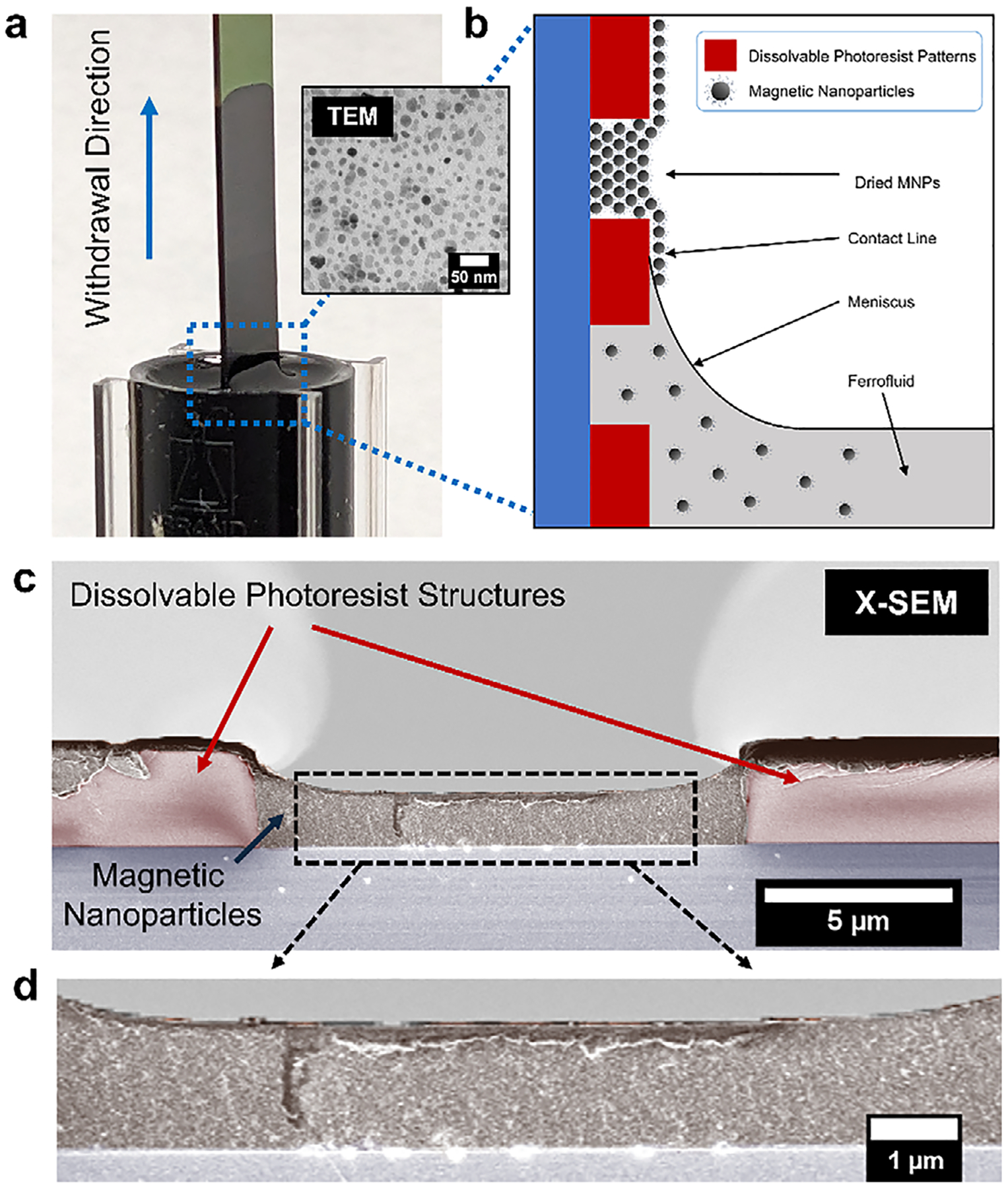
(a) Image of substrate being withdrawn from ferrofluid solution. For scale, the container holding the black ferrofluid is 12 mm in diameter. The inset shows a TEM image of the iron oxide nanoparticles. (b) Schematic showing the dip-coating process and deposition of MNPs on top of dissolvable photoresist micropatterns. (c) Cross-sectional scanning electron microscope (X-SEM) image showing dip-coated MNPs deposited on top of photoresist templates. (d) Magnified image showing region filled with MNPs. The image has false coloring to match the schematic in (b).

During the dip-coating process, a silicon wafer with patterned photoresist was submerged in a bath of MNP solution and withdrawn at a controlled speed. The MNP solution was a water-based ferrofluid (from Ferrotec), containing 15 ± 6 nm iron oxide nanoparticles that were coated with an anionic surfactant. A programmable dip-coater device was used to control the insertion speed, dwell time, and withdrawal speed during dip-coating deposition experiments. Figure 1a shows a picture of the dip-coating process. The substrate was withdrawn from the ferrofluid, which had a black color from the dispersion of iron oxide particles in solution. The inset shows a TEM image of the nanoparticles. The evaporation of particles on the surface of the substrate created a thin film of iron oxide MNPs. Figure 1b shows a schematic of the dip-coating process. As the substrate was pulled from solution, MNPs were deposited at the contact line. Selective deposition and filling within patterns were observed. Figure 1c shows a cross-sectional SEM image of a patterned substrate that has been withdrawn from ferrofluid solution. The dried MNPs formed a several-micrometer-thick film within the gaps between patterned photoresist. There was a thinner layer (several hundred nanometers) of MNPs deposited on top of the photoresist. The deposition within the gaps is consistent with discontinuous dewetting, a process where liquid selectively fills micropatterned wells as they are withdrawn from solution [23]. Video microscopy experiments (see Video S1 and Section S4) showed that deionized water selectively filled the photoresist patterns during the dewetting process.

**FIGURE 2 |:**
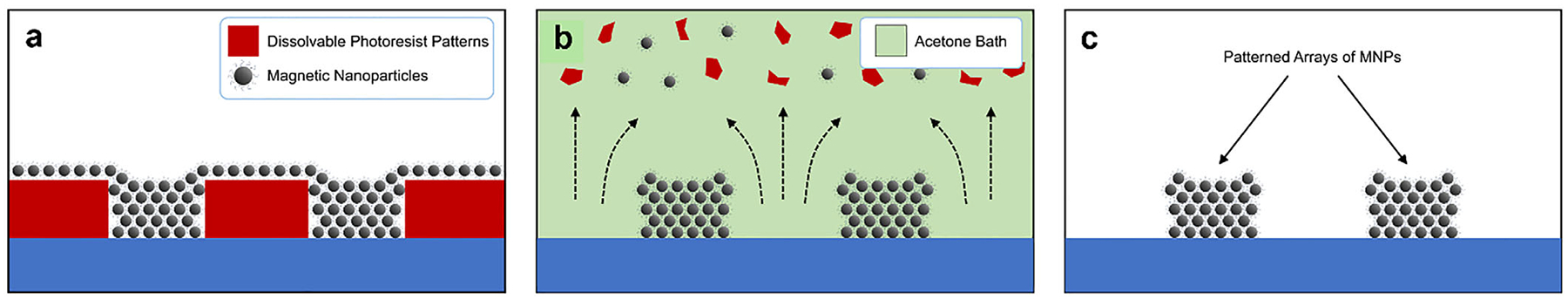
Process flow for dissolution of dissolvable templates and development of MNP micropatterns. (a) The process starts with a substrate that has been patterned with photoresist and dip-coated with MNPs. (b) The substrate is submerged in an acetone bath and gently ultrasonicated to dissolve the photoresist. (c) The dried MNP assemblies adhere to the substrate in patterned gaps between the photoresist.

After the MNPs were deposited onto the photoresist templates, the photoresist could be dissolved away to produce isolated assemblies of MNPs. Figure 2 shows a schematic of the process. First, the MNPs were deposited on a substrate that had been patterned with photoresist (Figure 2a). Then, the photoresist was dissolved using gentle ultrasonication in an acetone bath (Figure 2b). A variable transformer was used to control the power of ultrasonication so that the photoresist was dissolved, but the MNPs were not damaged. Finally, the substrate was rinsed with acetone and isopropyl alcohol and then dried with nitrogen gas. This process produced isolated assemblies of MNPs on the substrate (Figure 2c). For certain combinations of dip-coating parameters, pattern geometry, and development conditions, the yield across areas of several square centimeters ranged from 98.7% to 99.9% (Figure S2). For small features (10 μm and less), the sonication during development could delaminate the patterned structures, leading to a reduced yield. In one experiment, 20 and 10 μm disks were patterned on the same substrate and subjected to the same development conditions. The 20 μm disks had a significantly higher yield (98.8%) than the 10 μm disks (82.7%), which showed missing features from delamination (Figure S3).

**FIGURE 3 |:**
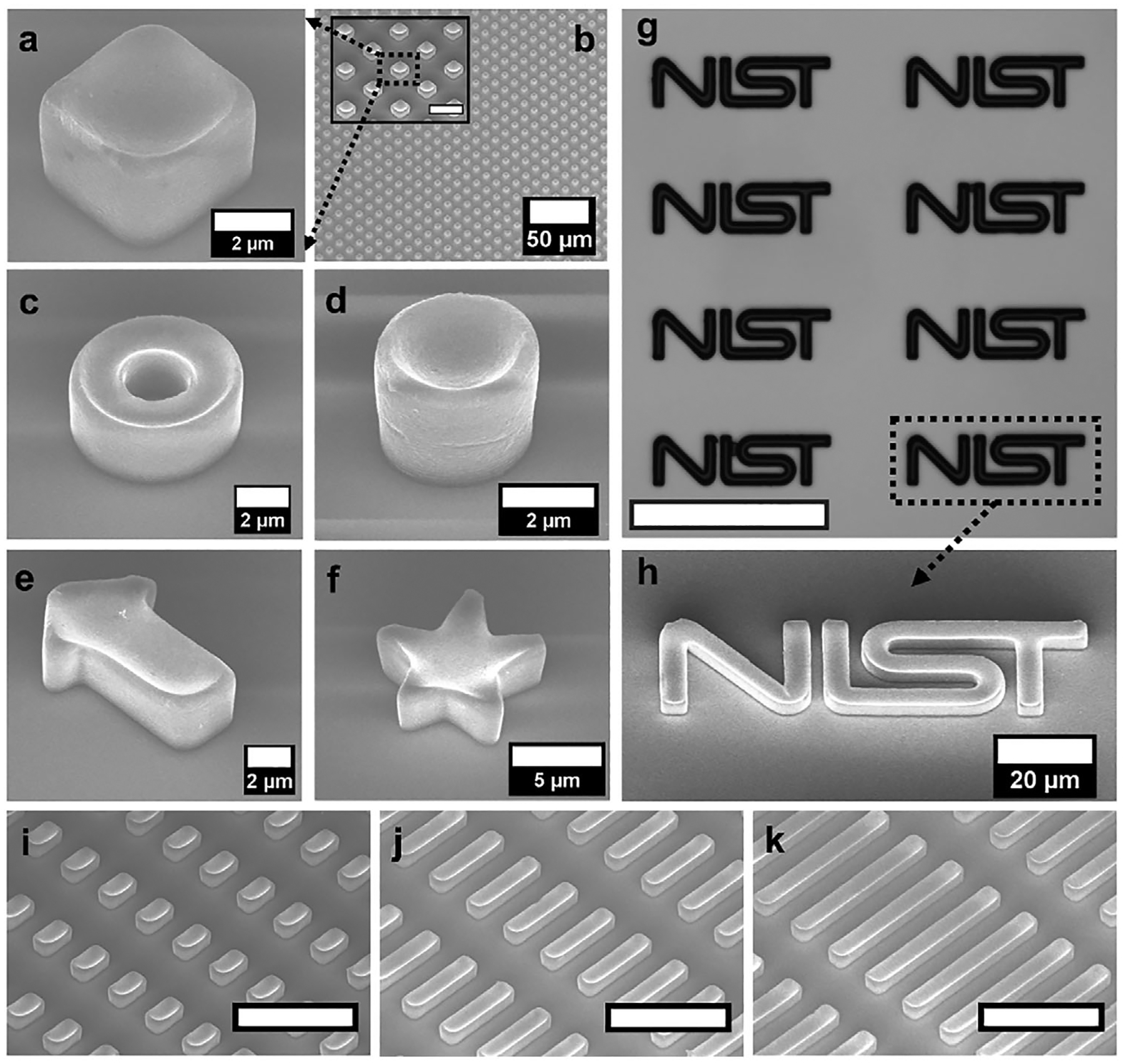
Examples of patterns produced using the template-directed dip-coating of MNPs. Tilt SEM images showing (a) a cuboid structure from (b) an array of cuboids (inset shows several patterned features; inset scale bar is 10 μm). Several shapes, including (c) a ring, (d) a pillar, (e) an arrow, and (f) a star. The technique was used to create an array with lettering, seen in (g) optical micrographs (scale bar is 100 μm) and (h) tilt SEM images. (i–k) Tilt SEM of bars with varying lengths (scale bars are 20 μm).

Figure 3 shows examples of MNP assemblies with well-defined geometries that were produced using the technique. The patterning resolution was limited only by the resolution of the original photolithography patterns (limited by optical diffraction). Figure 3a,b shows tilt SEM images of arrays of cuboid patterns. Figure 3b shows that the array was patterned across several hundred micrometers; although patterns across larger areas (up to several cm^2^) were possible. Figure 3c–f shows tilt SEM images of several structures produced by the process. The shapes include rings, pillars, arrows, and stars. The technique was also used to produce lettering and more sophisticated geometries. Figure 3g shows an optical microscope image of an array of lettering. Figure 3h shows a single set of letters observed using tilt SEM. The technique was also used to produce bar-shaped MNP assemblies with different aspect ratios (Figure 3i–k).

### 3.2 | MultiStep Patterning Schemes

**FIGURE 4 |:**
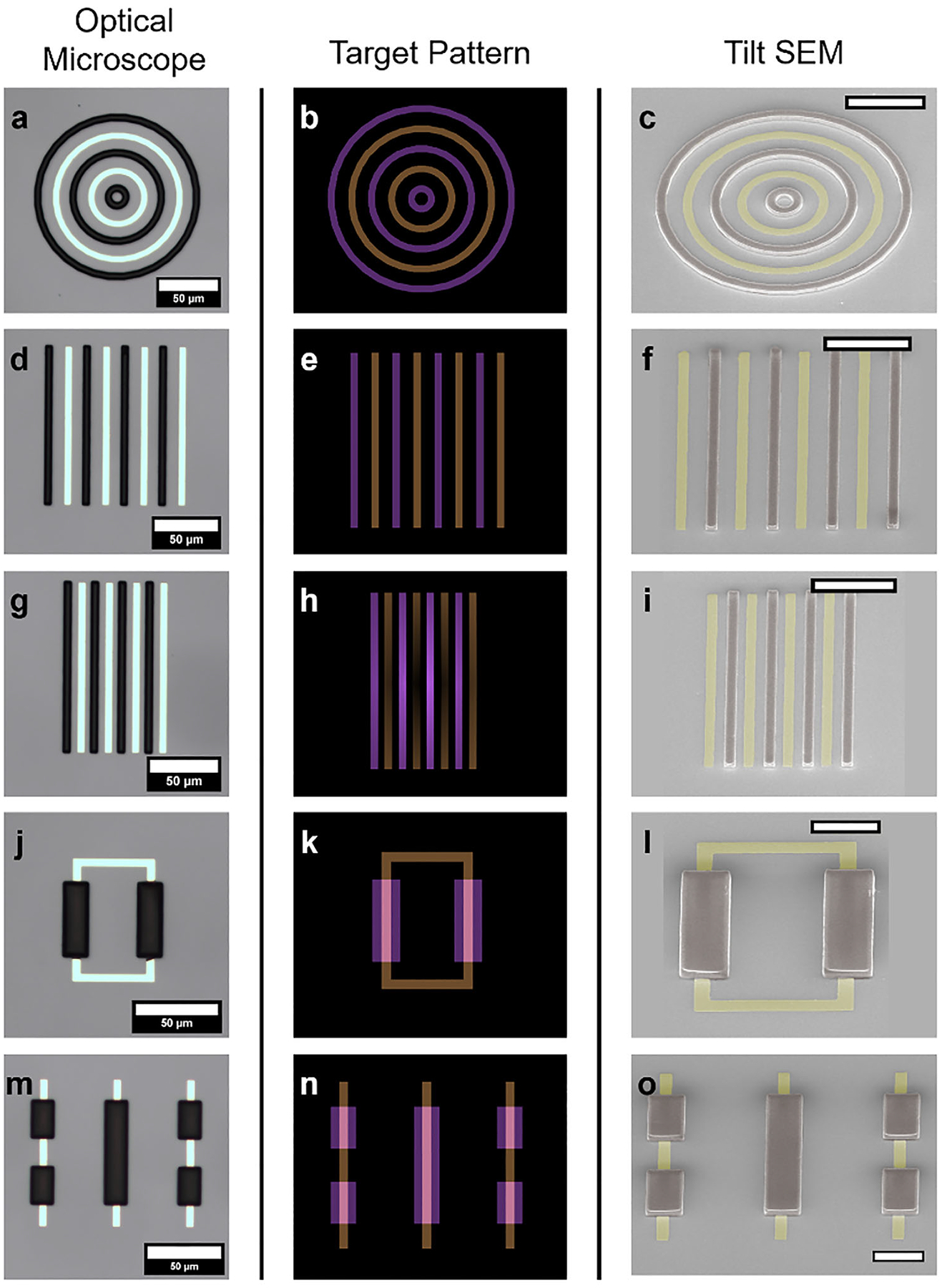
Comparison of optical microscope (a, d, g, j, m), original design schematics (b, e, h, k, n), and tilt SEM images (c, f, i, l, o) for co-patterned MNP arrays and gold thin films. False color was added to the tilt SEM images to differentiate between the MNPs (dark gray) and gold (light yellow). The scale bar in the tilt SEM images is 40 μm for (c), (f), (i), and (o), and 20 μm for (l).

The process was compatible with multistep lithography schemes, where MNP assemblies were patterned next to or on top of other types of materials. As an example, MNP assemblies were patterned next to micropatterned gold films. Figure 4 shows optical microscope images (first column; Figure 4a,d,g,j,m), target patterns (second column; Figure 4b,e,h,k,n), and tilt SEM images (third column; Figure 4c,f,i,l,o) of MNP structures that were interleaved with or patterned on top of gold thin films. For this process, the gold films were patterned first using a combination of photolithography and gold evaporation. After the gold films were created, a new layer of photoresist was used to create templates for dip-coating. After dip-coating and dissolution of the photoresist, MNP assemblies were fabricated in specific locations relative to the gold films. Using this procedure, patterns with interleaving rings and bars were created (Figure 4a–i). It was also possible to fabricate the MNP arrays directly on top of the micropatterned gold films (Figure 4j–o).

### 3.3 | Magnetometry

**FIGURE 5 |:**
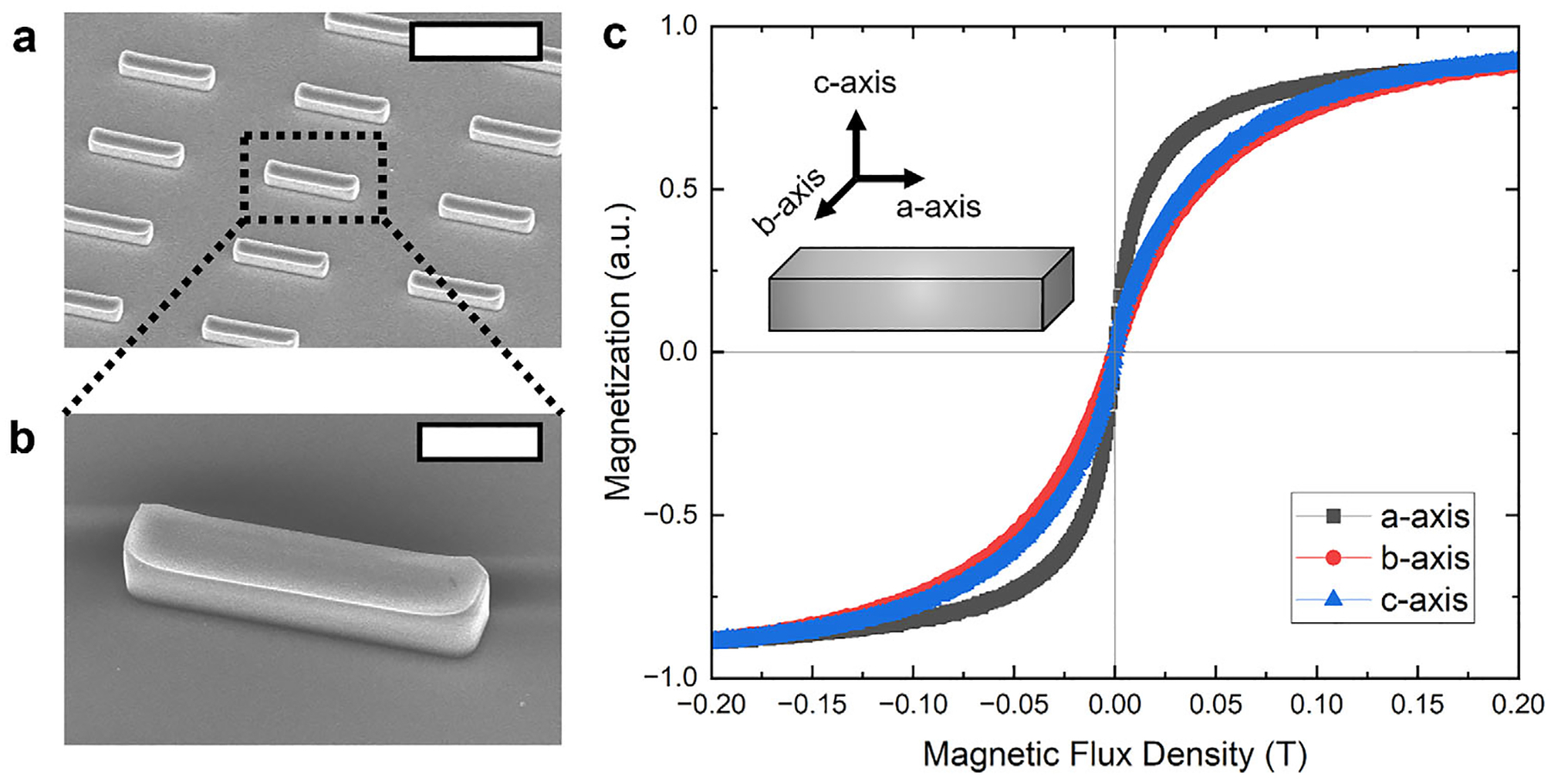
(a) SEM images of an array of micropatterned bars (scale bar is 20 μm). (b) Magnified image of a single patterned element (scale bar is 5 μm). (c) Normalized magnetization as a function of magnetic flux density for patterned assemblies along three axes.

The magnetic response of the MNP assemblies was influenced by their geometry. Figure 5a,b shows SEM images of an array of barshaped MNP micropatterns. The bars had dimensions of 15 μm along the *a*-axis, 3 μm along the *b*-axis, and 2–3 μm along the *c*-axis (orientations are labeled in Figure 5c). The edge-to-edge distance of the patterned features was 14 μm along both the *a*-axis and *b*-axis. The samples were patterned on a silicon substrate and measured with VSM along different orientations. Each orientation showed an “S” shaped magnetization vs. magnetic flux density curve, consistent with nanoscale superparamagnetic iron oxide nanoparticles [24]. However, the magnetic susceptibility of the micropatterns changed depending on their orientation to the applied magnetic field (Figure 5c). The orientation dependence of the magnetic susceptibility is a byproduct of the anisotropic shape of the micropatterns. The *a*-axis orientation, which also corresponded to the longest dimension of the bars, showed a higher magnetic susceptibility than the *b*-axis and *c*-axis. The effect can be understood in terms of demagnetization factors, *N*. The demagnetization factor along the *a*-axis, *N*_*a*_, was lower than the demagnetization factors along the *b*-axis and c-axis, because the patterned shapes were longest in that direction. The demagnetization factors for the *b*-axis and *c*-axis were approximately the same, *N*_*b*_ ~ *N*_*c*_, by symmetry. The magnetic particles in each patterned element experienced an internal field, *H*_*int*_, that was modified by a demagnetization correction, *H*_*int*_ = *H*_*appl*_ – *NM*. Since *N*_*a*_ < *N*_*b*_ ~ *N*_*c*_, the magnetic particles had the smallest demagnetization corrections for the *a*-axis, and, therefore, experienced the largest internal magnetic fields when oriented along the *a*-axis.

## 4 | Discussion

Over the past several decades, template-assisted assembly has emerged as a powerful technique for patterning nano- and microparticles [25–28]. When depositing particles from solution, templates can be used to control fluid dynamical forces and confine particles to produce well-defined colloidal structures [29, 30]. Dip-coating, on the other hand, is a mature technology (it is one of the oldest commercially applied thin film deposition techniques [31]) that is typically used to create continuous thin films. By using templated substrates and dip-coating in combination with one another, geometrically patterned particle assemblies can be created [32, 33].

The distinguishing feature of this work is the fabrication of micrometer-thick MNP assemblies with top-down engineered shapes and substrate locations. These relatively thick, patterned structures may be useful for the fabrication of microinductors and high-frequency electronics components. For electronics applications, it is advantageous to pattern magnetic materials next to or onto conducting layers. In a demonstration, MNP micropatterns were colocalized with prepatterned gold features (Figure 4), showing that MNP arrays can be patterned next to conducting materials using multistep alignment and lithography processes.

Additionally, arrays of shape anisotropic MNP composites showed an orientation-dependent magnetization in response to applied magnetic fields. This suggests that micropatterned MNP composites could be used to program the magnetic susceptibility and anisotropy of hierarchical materials, where the net magnetic properties are controlled by the microscale geometry of patterned MNPs. The programmed magnetic response could be used for applications in directed-assembly of magnetic materials or field-driven response of magnetic machines [34–38].

Dip-coating was chosen for this approach because it reproducibly created micrometer-thick films of MNPs. However, other types of deposition may also be compatible with the lift-off scheme for MNP patterning. For instance, spray pyrolysis has been used to micropattern silver nanoparticle films with a bilayer lift-off technique [39]. Dip-coating, however, has the added benefit of selective deposition within patterned wells. This selective deposition is consistent with discontinuous dewetting, which is caused by the withdrawal of micropatterned wells from solution [23]. Dip-coating is also relatively inexpensive compared to more complex forms of thin film deposition.

Dip-coating has other advantages, too. It can be used with a variety of substrates and can be scaled up to industrial-level production. It is also compatible with other types of nanomaterials, besides MNPs. It should be possible to deposit combinations of materials, so long as the two colloidal materials can be mixed to form the dip-coating solution. For example, quantum dots could be mixed with MNPs to form combinatorial structures with engineered magnetic and photonic properties.

In the experiments here, the micropatterned composites were held together by interparticle adhesion, presumably caused by van der Waals forces, after the solvent had dried away. For future technological applications, the mechanical strength of the composites could potentially be improved by crosslinking [17] or electro-infiltration [14, 15]. Alternatively, a protective material could be deposited on top of the structures.

## 5 | Conclusion

This article presents a method for fabricating geometrically precise MNP superstructures. The technique uses a combination of dip-coating and lift-off with dissolvable photoresist templates. MNP structures with micrometer-thickness with well-defined shapes, sizes, and spacings were produced. Possible applications include microinductors, magnetic microparticles for bioimaging, and magnetic materials with programmed properties.

## Supplementary Material

Supp1

Supp2

Additional supporting information can be found online in the Supporting Information section.

**Supporting Information File 1:** nano70101-sup-0001-VideoS1.mp4

**Supporting Information File 2:** nano70101-sup-0002-SuppMat.docx

## Figures and Tables

**TABLE 1:** Dip-coating parameters for micropatterns displayed in the article.

Figure	Immersion speed (mm/s)	Dwell time (s)	Withdrawal speed (mm/s)
1c	5	10	0.05
3	5	10	0.1
4	5	10	0.2
5	5	10	0.1

## Data Availability

The data that support the findings of this study are openly available in the NIST Public Data Repository at doi:10.18434/mds2-3869.
