# Supplementary material for: Microscale Assemblies of Magnetic Nanoparticles Produced by Dip-Coating and Lift-Off With Dissolvable Templates
Source: Nano Sel. Author manuscript; Available in PMC 2026 Jun 27. (PMC13307239; doi:10.1002/nano.70101)
Supplement: Supp1 [file NIHMS2183647-supplement-Supp1.docx]

Supporting Information for “Microscale Assemblies of Magnetic Nanoparticles Produced by Dip-Coating and Lift-Off with Dissolvable Templates”

Samuel D. Oberdick*, Alexey V. Nazarov, Gary Zabow

S. D. Oberdick

Department of Physics, University of Colorado, Boulder, Colorado 80309, USA.

National Institute of Standards and Technology, Boulder, Colorado 80305, USA.
E-mail: samuel.oberdick@nist.gov

A. V. Nazarov, G. Zabow

National Institute of Standards and Technology, Boulder, Colorado 80305, USA.

Contents

Section S1. Microfabrication Details

Section S2. Size Distribution of Nanoparticles

Section S3. Yield Analysis

Section S4. Video Microscopy of Dewetting on Micropatterns

S1. Microfabrication Details

All microfabrication supplies, such as wafers, and chemicals, such as photoresist and developers, were supplied by the microfabrication facility at the National Institute of Standards and Technology (NIST) in Boulder, Colorado.

Silicon wafers with a diameter of 76.2 mm and a thickness of 380 μm were used as substrates for the patterned photoresist. Wafers were first cleaned using a solvent rinse (acetone and isopropyl alcohol) and an oxygen plasma (60 W, 50 sccm, 120 s). In a typical process, an automated spin-coater was used to spin-coat (4 kRPM spin speed, 40 s spin time) an adhesion promoter (P-20 hexamethyldisilazane, or HMDS, from Transene) onto the silicon wafer. Then, photoresist (Megaposit SPR 220-3) was spun (2 kRPM spin speed, 45 s spin time) on top of the adhesion promoter. The substrate was baked at 115 °C for 95 s (90 s in vacuum mode and 5 s in contact mode). The photoresist was exposed using a Heidelberg MLA 150 maskless aligner system. The substrate was hand developed (Megaposit MF 26A developer) for 45 s to 55 s, depending on the pattern. The substrate was cleaned using a spin/rinse/dry cycle. Finally, the substrate was exposed to an oxygen plasma (60 W, 50 sccm, 60 s) to promote wetting during the dip-coating process.

The gold thin film micropatterns were fabricated using electron beam evaporation and lift-off. An automated spin-coater was used to spin-coat (4 kRPM spin speed, 45 s spin time) an adhesion promoter (P-20 hexamethyldisilazane, or HMDS, from Transene) onto the silicon wafer. Then, photoresist (Kayaku/Megaposit SPR 660) was spun (2.2 kRPM spin speed, 35 s spin time) on top of the adhesion promoter. The substrate was baked at 95 °C for 60 s (in vacuum mode). The photoresist was exposed using a Heidelberg MLA 150 maskless aligner system. The substrate was baked again (post-exposure bake) at 110 °C for 60 s. The substrate was hand developed (Megaposit MF 26A developer) for 30 s. Gold was evaporated (electron beam evaporation system from AJA International Inc.) on top of the patterned photoresist. The gold was deposited at a rate of 0.1 nm/s. The final thickness of the gold was 50 nm. Lift-off was performed by sonicating the substrate in acetone for 90 s (70% power, controlled using a variable transformer). Sonication removed the photoresist, leaving behind micropatterned gold thin film features. After the gold layer was patterned, photoresist templates for dip-coating were fabricated on top of the gold using the alignment feature of the maskless aligner system.

S2. Size Distribution of Nanoparticles

Transmission electron microscopy (TEM) was performed using a FEI Tecnai T12 Spirit BT microscope (120 kV, LaB_6_). Images were analyzed using ImageJ^[1]^ to determine particle size distribution (Figure S1).

Figure S1. (a) Transmission electron microscope (TEM) image of iron oxide nanoparticles. (c) Size distribution of particle diameters measured from TEM image.


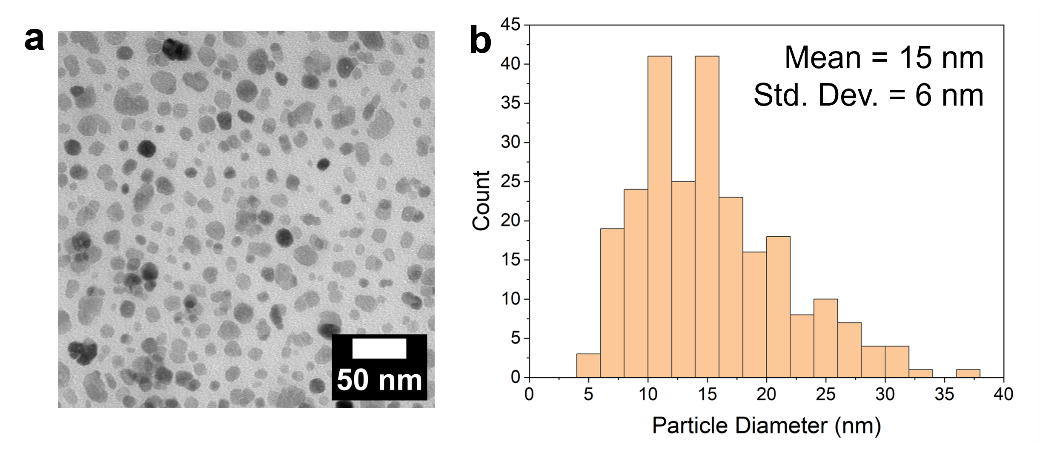


S3. Yield Analysis

When experimental conditions are optimized, it is possible to achieve nearly 100% micropatterning yield across relatively large areas. Figure S2 shows the yield across several square millimeters for a pattern consisting of 20 μm diameter circles. The dip-coating parameters were: 5 mm/s insertion speed, 10 s dwell time, and 0.1 mm/s withdrawal speed. The defects in the magnified regions of Figure S2 were manually counted to determine the yield.

Figure S2. Large area optical microscope image of silicon substrate patterned with 20 μm diameter disks. Magnified regions across the substrate show yields of 98.7% to 99.9%.


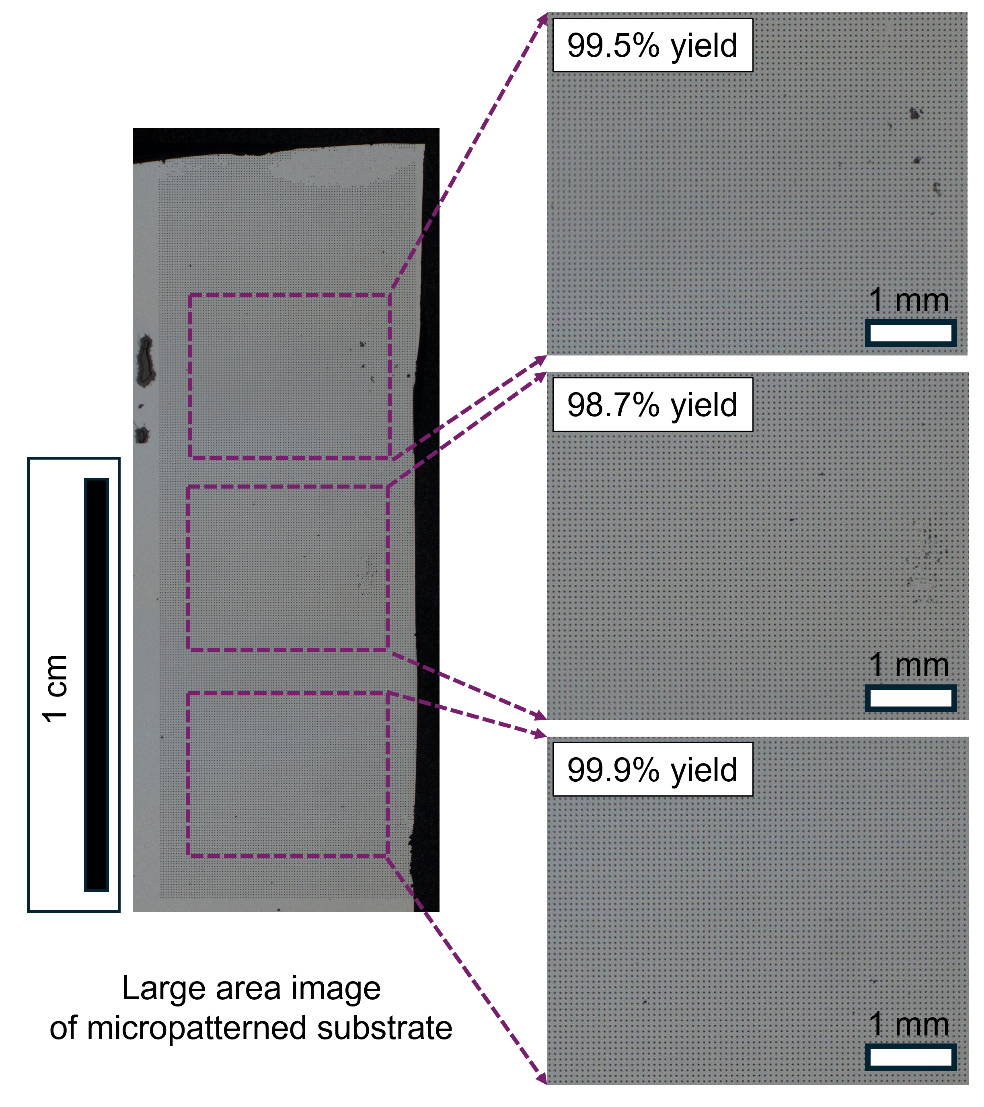


Smaller features could delaminate using development conditions that worked well for larger features. Figure S3 shows a substrate with two feature types, 10 μm and 20 μm disks. The 20 μm disks had a higher yield (98.8%) than the 10 μm disks (82.7%). The dip-coating parameters were: 5 mm/s insertion speed, 10 s dwell time, and 0.1 mm/s withdrawal speed. For Figure S3, images were analyzed using ImageJ^[1]^ to determine the yield.

Figure S3. Large area optical microscope image of silicon substrate patterned with 10 μm (left) and 20 μm (right) diameter disks. The 20 μm disks had a significantly higher yield than the 10 μm disks.


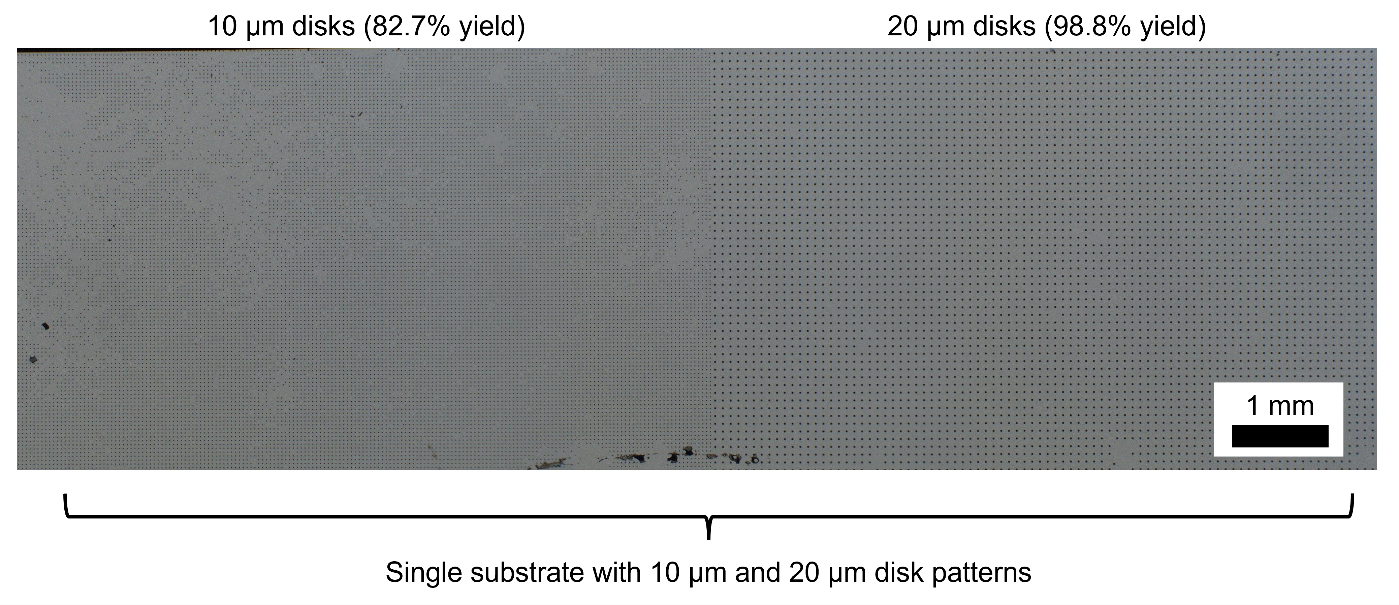


S4. Video Microscopy of Dewetting on Micropatterns

The dewetting of deionized water from a vertically mounted micropatterned sample was characterized using video microscopy. For these experiments, a drop of water was deposited onto the surface of the substrate and then pipetted away to induce dewetting. The dip-coating apparatus was not used because of limited optical access for video recording. The experiments showed that water was deposited within the patterned structures as the liquid contact line moved across the field of view, consistent with discontinuous dewetting. After the water was deposited within the patterns, it evaporated. The video is included in the supporting information. The field of view in the vertical direction is approximately 200 μm. The video is slowed down by 0.25x compared to normal speed. The dewetting contact line moves at a speed of approximately 0.1 mm/s, similar to the dip-coating withdrawal speeds used for experiments in the main manuscript.

**Disclaimer**

Certain equipment, instruments, or materials are identified in this paper in order to specify the experimental procedure adequately.  Such identification is not intended to imply recommendation or endorsement of any product or service by NIST, nor is it intended to imply that the materials or equipment identified are necessarily the best available for the purpose.

**References**

[1] C. A. Schneider, W. S. Rasband, K. W. Eliceiri, *Nat. Methods* 2012, *9*, 671.
